# Hematological ratios, immune-related adverse events and mortality in patients treated with immune checkpoint inhibitors

**DOI:** 10.1371/journal.pone.0336491

**Published:** 2025-11-12

**Authors:** Sophie Lekkerkerker, Karin A. H. Kaasjager, Saskia Haitjema, Cornelia Hulsbergen-Veelken, Karin H. Herbschleb, Marianne C. Verhaar, Meriem Khairoun, Gurbey Ocak

**Affiliations:** 1 Department of Internal Medicine, Sint Antonius Hospital, Nieuwegein, The Netherlands; 2 Department of Nephrology and Hypertension, University Medical Center Utrecht, Utrecht, The Netherlands; 3 Department of Internal Medicine and Dermatology, University Medical Center Utrecht, Utrecht, The Netherlands; 4 Central Diagnostic Laboratory, University Medical Center Utrecht, Utrecht, The Netherlands; Cleveland Clinic, UNITED STATES OF AMERICA

## Abstract

**Background:**

Although immune checkpoint inhibitors improve survival in patients with malignancies, a substantial number of patients treated with these agents experience immune-related adverse events. It is unknown whether inflammation-related hematological ratios are associated with immune-related adverse events or mortality.

**Objective:**

We aimed to investigate the association between pretreatment inflammation-related hematological ratios and the occurrence of immune-related adverse events and mortality in patients receiving checkpoint inhibitors.

**Methods:**

Patients treated with checkpoint inhibitors within a tertiary hospital in the Netherlands were studied using routine care data between January 2013 and May 2020. Cox regression analysis was performed to assess the association between neutrophil-to-lymphocyte ratio (NLR), platelet-to-lymphocyte ratio (PLR), neutrophil-to-lymphocytes and platelets ratio (NLPR), and systemic immune-inflammation index (SII) and outcomes (immune-related adverse events or mortality).

**Results:**

Among 664 patients treated with checkpoint inhibitors, 397 (59.8%) patients developed an immune-related adverse event and 363 (54.7%) patients died during a median follow-up period of 17 months (interquartile range 7–30 months). Hematological ratios were not associated with immune-related adverse events. However, highest tertiles as compared with lowest tertiles of all hematological ratios were independently associated with mortality (NLR: adjusted hazard ratio (HR) 2.23, 95% CI 1.69–2.95; PLR: adjusted HR 1.88, 95% CI 1.43–2.47; NLPR: adjusted 1.59, 95% CI 1.22–2.06; SII: adjusted HR 2.33, 95% CI 1.77–3.08).

**Conclusion:**

In this study, pretreatment inflammation-based hematological ratios were not associated with future immune-related adverse events in patients treated with checkpoint inhibitors. However, elevated hematological ratios were associated with an increased mortality risk.

## Introduction

The development of immune checkpoint inhibitors (ICPi) have been a breakthrough in the field of medical oncology [1,2]. ICPi are humanized monoclonal antibodies that block inhibitory checkpoints in T-cells, hereby stimulating the immune system to target malignancies [3,4]. Besides the remarkable clinical benefits, ICPi have been associated with immune-related adverse events (irAE). The exact pathophysiology of irAEs across different organ systems is not yet fully clarified, but contributing factors include latent autoimmunity triggered by ICPi therapy, the presence of similar antigens on both tumor and normal tissue, increased inflammation and reduced self-tolerance [5–8]. IrAE are reported in up to 71% of patients who are treated with ICPi, and most frequently involve the gastrointestinal tract, liver, skin, and endocrine system [9]. Although the majority of irAE are mild and reversible, life-threatening and permanent events are not uncommon [10]. Early recognition and initiation of treatment, usually in the form of immunosuppression, are critical to reduce the risk of poor outcome [11]. However, differentiating irAE from other diseases or progressive disease can be challenging due to similarities in clinical presentation [12].

A major challenge remains the identification of patients who are susceptible to the development of irAE before the start of ICPi treatment. Immunotherapy is expanding its indications for patients without metastatic disease across various cancer types, including colorectal cancer and non-small cell lung cancer. Consequently, immune-related adverse events (irAEs) are becoming increasingly relevant when weighing risks and benefits in healthier populations as compared with patients with metastatic disease. The identification of risk factors for irAE could help patient selection for ICPi treatment and improve early irAE detection through personalized monitoring. Therefore, the identification of risk factors for irAE has become an area of growing interest [13]. Several studies have investigated potential biomarkers, including specific autoantibodies [14], the intestinal microbiome [15], genetic predisposition [16], albumin and thyroid-stimulating hormone [17]. However, these markers are not used in clinical practice to identify patients who are susceptible to the development of irAE before the start of ICPi treatment.

Several hematological ratios that can be derived from complete blood count are known to reflect systemic inflammatory status [18–23]. As inflammation is thought to play an important part in the development of irAE, inflammation-related hematological ratios could be risk factors for irAE in patients treated with ICPi. Indeed, a retrospective study found an association between two hematological ratios, the neutrophil-to-lymphocyte ratio (NLR) and platelet-to-lymphocyte ratio (PLR), and occurrence of irAE in patients receiving ICPi for advanced non-small-cell lung carcinoma [24]. The systemic immune-inflammation index (SII) has been investigated as a potential risk indicator for immune-related adverse events (irAEs) in certain tumors, including liver cancer and metastatic urothelial carcinoma [25,26]. The neutrophil-to-lymphocyte ratio (NLPR) has not yet been explored as a potential risk factor for the prediction of irAEs [27,28]. As complete blood count is already part of routine blood sampling in the recommended before the start of ICPi treatment [29], these ratios could be clinically convenient and inexpensive biomarkers. Furthermore, it could be that these hematological ratios could be of prognostic value in the prediction of mortality in patients with ICPi use.

The aim of this study was to determine the association between pretreatment inflammation-related peripheral blood ratios (NLR, PLR, NLPR, and SII) and the occurrence of irAE and mortality in patients with cancer treated with ICPi.

## Methods

### Study design and population

This observational cohort study was performed in patients aged ≥ 18 years who were treated with ICPi within the University Medical Center Utrecht, a tertiary academic hospital in the Netherlands. Patients were included if they had received at least one dose of nivolumab, ipilimumab, pembrolizumab, atezolizumab, durvalumab, tremelimumab, or a combination of them, between January 1, 2013 and May 31, 2020.

The study was performed in accordance with the Declaration of Helsinki. The institutional ethical review board decided that the Medical Research Involving Human Subjects Act did not apply to this study as no patients were subjected to interventions. Therefore, an official approval of the board was not required (METC number 20–312/C).

### Data collection

Patients who had received ICPi in the abovementioned study period were identified by searching the hospital’s registry of oncology medication. The identified patient IDs were then connected to the Utrecht Patient Oriented Database (UPOD), a database that contains demographics, laboratory measurements, and medication data of all patients treated at our hospital. The structure and content of the UPOD have been described elsewhere [30].

Extracted data from the UPOD included demographics, complete blood counts, body mass index, use of antihypertensive and immunosuppressive medication and prior treatment with chemotherapy. Data on characteristics and treatment of irAE, comorbidities including autoimmune disease and smoking status were manually extracted from the electronic health records by two researchers. If patients consecutively developed more than one irAE, only the first episode of an irAE was recorded and analyzed. Comorbidities were quantified using the Charlson Comorbidity Index [31].

Patients were recorded as having hypertension if they received antihypertensive medication at the date of first ICPi administration. Outcome data were collected up to July 31, 2021.

### Hematological ratios

The hematological ratios were derived from the most recent peripheral blood sample up to six weeks before first ICPi administration. The median period between the most recent peripheral blood sample and first ICPi administration was 1 (IQR 0–5) day. The neutrophil-to-lymphocyte ratio (neutrophil count/ lymphocyte count), platelet-to-lymphocyte ratio (platelet count/ lymphocyte count), neutrophil-to-lymphocytes and platelets ratio (neutrophil count × 100)/ (lymphocyte count x platelet count)), and systemic immune-inflammation index (platelet count x (neutrophil count/ lymphocyte count)) were calculated from complete blood counts. These ratios were further categorized into tertiles, with the lowest tertile used as the reference group. For NLR, the lowest tertile was less than 2.63, the middle tertile was between 2.63 and 4.68, and the highest tertile was above 4.68. For PLR, the lowest tertile was less than 143.38, the middle tertile was between 143.38 and 246.51, and the highest tertile was above 246.51. For NLPR, the lowest tertile was less than 0.94, the middle tertile was between 0.94 and 1.59, and the highest tertile was above 1.59. For SII, the lowest tertile was less than 666.57, the middle tertile was between 666.57 and 1455.06, and the highest tertile was above 1455.06.

### Outcomes

The primary outcome was a first episode of an irAE. An irAE was defined as any event referred to as immune-related toxicity by the treating physician. Reactions and exacerbations of pre-existing autoimmune disease were not included in the irAE definition. IrAE were graded according to the Common Terminology Criteria for Adverse Events (CTCAE) version 5.0 [31]. The secondary outcome was all-cause mortality as registered in the electronic health record.

### Statistical analysis

Baseline characteristics of the study population were described using medians and interquartile ranges for continuous variables and counts and percentages for categorical variables. Cox regression analysis was performed to calculate crude and adjusted hazard ratios (HRs) with 95% confidence intervals (CIs) to explore the association between hematological ratios and outcomes (first episode of irAE and mortality).

Hazard ratios were adjusted for potential confounders including age, sex, malignancy type, body mass index, Charlson Comorbidity Index, hypertension, smoking status, prior chemotherapy, immunosuppressive medication, and history of autoimmune disease. Hematological ratios were divided into tertiles, with the lowest tertile used as reference. Furthermore, HRs were calculated by using hematological ratios as continuous factors in the Cox regression models. The proportional hazards assumption was evaluated by use of a log–log plot, and formally tested by the use of Schoenfeld residuals.

All analyses were performed using IBM SPSS Statistics version 25.0.

## Results

### Baseline characteristics

A total of 678 patients received ICPi treatment within the study period. After exclusion of 14 patients who had objected to use of their data for research purposes, 664 patients were included in the analysis. Baseline characteristics of the included patients are shown in Table 1. The median age was 64 (IQR 54–71) years and 415 (62.5%) patients were male. Melanoma (47.4%) was the most frequent malignancy type, followed by non-small cell lung carcinoma (24.2%) and urinary tract cancer (11.9%). Pembroluzimab monotherapy was the most commonly prescribed ICPi, used by 233 (35.1%) patients. Seventeen patients had a history of autoimmune disease, while 114 patients were using immunosuppressive medication. In the majority of cases, these immunosuppressive medications were prescribed to reduce symptoms related to tumor activity.

**Table 1:** Baseline characteristics of the study population by irAE status.

	Total (N = 664)		No irAE (N = 267)		irAE (N = 397)	
Age (years), median (IQR)	64	(54-71)	63	(53-70)	64	(54-72)
Sex (male), N (%)	415	(62.5)	179	(67.0)	236	(59.4)
Cancer type, N (%)						
Melanoma	315	(47.4)	95	(35.6)	220	(55.4)
NSCLC	161	(24.2)	64	(24.0)	97	(24.4)
Urinary tract	79	(11.9)	50	(18.7)	29	(7.3)
Gynecologic	19	(2.9)	4	(1.5)	15	(3.8)
Hematologic	35	(5.3)	22	(8.2)	13	(3.3)
Gastrointestinal	27	(4.1)	19	(7.1)	8	(2.0)
Head and neck	18	(2.7)	10	(3.7)	8	(2.0)
Other	10	(1.5)	3	(1.1)	7	(1.8)
Charlson Comorbidity Index, median (IQR)	8	(7-9)	8	(7-9)	8	(7-9)
History of autoimmune disease, N (%)	17	(2.6)	5	(1.9)	12	(3.0)
Use of immunosuppressive medication, N (%)	114	(17.2)	50	(18.7)	64	(16.1)
Smoking status, N (%)						
Never	298	(44.9)	119	(44.6)	179	(45.1)
Former/current	363	(54.7)	147	(55.1)	216	(54.4)
Missing	3	(0.5)	1	(0.4)	2	(0.5)
BMI, median (IQR)	25.7	(23.4-28.6)	25.7	(23.3-27.9)	25.8	(23.4-28.8)
Hypertension, N (%)	229	(34.5)	86	(32.2)	143	(36.0)
Prior chemotherapy or targeted therapy, N (%)	191	(28.8)	101	(37.8)	90	(22.7)
Checkpoint inhibitor type, N (%)						
Nivolumab	198	(29.8)	95	(35.6)	103	(25.9)
Ipilimumab	45	(6.8)	22	(8.2)	23	(5.8)
Pembrolizumab	233	(35.1)	90	(33.7)	143	(36.0)
Durvalumab	20	(3.0)	5	(1.9)	15	(3.8)
Atezolizumab	33	(5.0)	20	(7.5)	13	(3.3)
Tremelimumab	6	(0.9)	2	(0.7)	4	(1.0)
Combined nivolumab and ipilimumab	129	(19.4)	33	(12.4)	96	(24.2)
Hematological ratios						
NLR, median (IQR)	3.5	(2.2-5.5)	3.8	(2.4-6.5)	3.3	(2.1-5.0)
PLR, median (IQR)	189	(129-284)	206	(132-335)	180	(127-269)
NLPR, median (IQR)	1.2	(0.8-2.0)	1.2	(0.9-2.2)	1.2	(0.8-1.8)
SII, median (IQR)	955	(567-1800)	1158	(570-2181)	857	(561-1584)

BMI, Body Mass Index; IQR, interquartile range; irAE, immune-related adverse event; NLPR, neutrophil-to-lymphocytes and platelets ratio; NLR, neutrophil-to-lymphocyte ratio; NSCLC, non-small-cell lung carcinoma; PLR, platelet-to-lymphocyte ratio; SII, systemic immune-inflammation index.

### Incidence and characteristics of immune-related adverse events

Among all patients analyzed in the study, 397 (59.8%) patients developed a first irAE following start of ICPi therapy (Table 2). The majority of irAE (75.5%) were grade 1 or 2. Dermatologic irAE were the most frequent (29.9%), followed by gastro-intestinal (21.4%) and endocrine irAE (17.4%). The median time from start of ICPi therapy to the onset of irAE was 8 (IQR 3–15) weeks. Most patients with irAE were treated with systemic corticosteroids (n = 142;35.8%) and 77 (19.4%) patients with irAE were treated with topical corticosteroids. In 81 (20.4%) patients with irAE other treatments were started, including biologicals (primarily infliximab for colitis), selective immunosuppressive drugs (primarily Mycophenolate Mofetil for hepatitis), hormonal replacement therapy and symptomatic therapy, e.g., analgesic medication and skin ointments.

**Table 2:** Characteristics of the first irAE.

All patients, N (%)	664	(100)
Patients with irAE, N (%)	397	(59.8)
IrAE grade, N (%)		(100)
Grade 1	147	(37.0)
Grade 2	153	(38.5)
Grade 3	71	(17.8)
Grade 4	10	(2.5)
Missing	16	(4.0)
Treatment, N (%)		
Systemic corticosteroids	142	(35.8)
Topical corticosteroids	77	(19.4)
Other	81	(20.4)
Affected organ system, N (%)		
Cardiovascular	2	(0.5)
Dermatologic	119	(29.9)
Endocrine	69	(17.4)
Gastro-intestinal	85	(21.4)
Hematologic	3	(0.7)
Musculoskeletal	35	(8.8)
Neurologic	9	(2.3)
Ocular	6	(1.5)
Pulmonary	33	(8.3)
Renal	9	(2.3)
Systemic	8	(2.0)
Nonspecific/Constitutional	19	(4.8)

irAE, immune-related adverse event.

### Hematological ratios and irAE

The association between the hematological ratios and irAE is shown in Table 3. In comparison with the lowest tertiles, the highest tertiles of the NLR, PLR, and NLPR were not associated with irAE in both the crude and adjusted Cox regression model as compared with the lowest tertiles. In a continuous analysis, also none of the hematological ratios were associated with irAE.

**Table 3:** Association between hematological ratios and occurrence of irAE.

		Hazard ratios for immune-related adverse event			
		Crude HR (95% CI)		Adjusted HR* (95% CI)	
NLR	Tertile 1	1.0	(reference)	1.0	(reference)
	Tertile 2	0.98	(0.77-1.23)	0.98	(0.77-1.24)
	Tertile 3	0.93	(0.73-1.19)	1.02	(0.79-1.32)
PLR	Tertile 1	1.0	(reference)	1.0	(reference)
	Tertile 2	1.15	(0.91-1.45)	1.20	(0.95-1.53)
	Tertile 3	0.91	(0.71-1.16)	1.03	(0.79-1.34)
NLPR	Tertile 1	1.0	(reference)	1.0	(reference)
	Tertile 2	0.79	(0.62-1.00)	0.80	(0.63-1.02)
	Tertile 3	0.93	(0.73-1.18)	1.00	(0.78-1.28)
SII	Tertile 1	1.0	(reference)	1.0	(reference)
	Tertile 2	**1.29**	**(1.02-1.63)**	**1.33**	**(1.05-1.69)**
	Tertile 3	0.98	(0.76-1.26)	1.08	(0.83-1.41)
Continuous hematological ratios					
NLR	(continuous)	0.987	(0.964-1.010)	0.993	(0.970-1.017)
PLR	(continuous)	0.999	(0.999-1.000)	1.000	(0.999-1.000)
NLPR	(continuous)	0.971	(0.923-1.022)	0.987	(0.939-1.038)
SII/1000	(continuous)	0.942	(0.889-1.008)	0.954	(0.889-1.024)

CI, confidence interval; HR, hazard ratio; NLPR, neutrophil-to-lymphocytes and platelets ratio; NLR, neutrophil-to-lymphocyte ratio; PLR, platelet-to-lymphocyte ratio; SII, systemic immune-inflammation index.

*Adjusted for age, sex, Charlson Comorbidity Index, hypertension, smoking status, BMI, malignancy type, prior chemotherapy, immunosuppressive medication, and history of autoimmune disease.

### Hematological ratios and mortality

During a median follow-up period of 17 (IQR 7–30) months, 363 (54.7%) patients died. Table 4 shows the association between the hematological ratios and mortality. After adjustment for potential confounders in the multivariate Cox regression model, including age, sex, malignancy type, body mass index, Charlson Comorbidity Index, hypertension, smoking status, prior chemotherapy, use of immunosuppressive medication, and history of autoimmune disease, the highest tertiles of the NLR (HR 2.23, 95% CI 1.69–2.95), PLR (HR 1.88, 95% CI 1.50–2.60), NLPR (HR 1.74, 95% CI 1.34–2.27) and SII (HR 2.33, 95% CI 1.77–3.08) were associated with an increased mortality risk. In a continuous model, NLR (HR 1.05, 95% CI 1.03–1.06), PLR (HR 1.002, 95% CI 1.001–1.003), and NLPR (HR 1.07, 95% CI 1.04–1.10) were also associated with an increased mortality risk.

**Table 4:** Association between hematological ratios and mortality.

		Hazard ratios for mortality			
		Crude HR (95% CI)		Adjusted* HR (95% CI)	
NLR	Tertile 1	1.0	(reference)	1.0	(reference)
	Tertile 2	**1.56**	**(1.18-2.06)**	**1.57**	**(1.18-2.07)**
	Tertile 3	**2.55**	**(1.95-3.33)**	**2.40**	**(1.83-3.17)**
PLR	Tertile 1	1.0	(reference)	1.0	(reference)
	Tertile 2	**1.46**	**(1.11-1.92)**	**1.44**	**(1.09-1.89)**
	Tertile 3	**2.15**	**(1.66-2.80)**	**1.88**	**(1.50-2.60)**
NLPR	Tertile 1	1.0	(reference)	1.0	(reference)
	Tertile 2	1.25	(0.95-1.63)	1.22	(0.93-1.60)
	Tertile 3	**1.89**	**(1.46-2.44)**	**1.74**	**(1.34-2.27)**
SII	Tertile 1	1.0	(reference)	1.0	(reference)
	Tertile 2	**1.39**	**(1.05-1.84)**	**1.39**	**(1.05-1.84)**
	Tertile 3	**2.61**	**(2.00-3.39)**	**2.52**	**(1.91-3.32)**
Continuous hematological ratios					
NLR	(continuous)	**1.053**	**(1.040-1.066)**	**1.052**	**(1.038-1.066)**
PLR	(continuous)	**1.002**	**(1.001-1.002)**	**1.002**	**(1.001-1.003)**
NLPR	(continuous)	**1.072**	**(1.046-1.099)**	**1.073**	**(1.043-1.104)**
SII/1000	(continuous)	**1.178**	**(1.133-1.225)**	**1.175**	**(1.127-1.224)**

CI, confidence interval; HR, hazard ratio; NLPR, neutrophil-to-lymphocytes and platelets ratio; NLR, neutrophil-to-lymphocyte ratio; PLR, platelet-to-lymphocyte ratio; SII, systemic immune-inflammation index.

*Adjusted for age, sex, Charlson Comorbidity Index, hypertension, smoking status, BMI, malignancy type, prior chemotherapy, immunosuppressive medication, and history of autoimmune disease.

## Discussion

In patients receiving ICPi treatment, the onset of irAE may be due to an imbalance in enhanced inflammatory response and decreased self-tolerance. Therefore, we studied the association between hematological biomarkers, including neutrophil-to-lymphocyte ratio (NLR), platelet-to-lymphocyte ratio (PLR), neutrophil-to-lymphocytes and platelets ratio (NLPR) and systemic immune-inflammation index (SII) and IrAEs or mortality in a cohort of 664 patients treated with ICPi. We found no significant association between hematological ratios and irAEs in patients receiving ICPi therapy. However, elevated NLR, PLR, NLPR and SII-index were associated with an increased risk of mortality.

### Hematological ratios and immune-related adverse events

The association between NLR and PLR and the development of irAEs has been investigated in previous studies [24,32–36]. The findings in our study are consistent with the findings in a retrospective review of patients with non-small cell lung cancer (NSCLC), which described no significant association between NLR, PLR and increased risk of irAEs [32]. In contrast to our study, other studies found an association between NLR, PLR [24,33–36] or SII [37] and the onset of irAE. There could be several reasons for the discrepancies between our study and previous studies [24,33–37], including differences in study populations, comorbidities, and differences in definitions and distribution of irAEs. Our study includes a patient population comprising various malignancies, including melanoma, lung cancer, and urinary tract cancers, while several previous studies solely focused on patients diagnosed with NSCLC [24,35,36]. Furthermore, our study encompassed a larger number of patients than previous studies [24,34]. Another explanation for the differences could be that less patients received chemotherapy or targeted therapy prior to immunotherapy in our study as compared with the other studies [24,36].

### NLR and mortality

Our results are in line with a meta-analysis indicating that a high baseline NLR is a significant predictor of worse overall and progression-free survival in patients with NSCLC, melanoma, and genitourinary cancers treated with immune checkpoint inhibitors [38]. Our findings supports this association, indicating that elevated pretreatment NLR is a potentially useful tool in selection and follow-up of patients receiving ICPi therapy. Furthermore, the NLR could be used to discuss prognosis and expectations of patients during therapy.

### PLR and mortality

In our analyses, we observed an elevated mortality risk associated with elevated PLR values, which is in line with the literature. A meta-analysis including 12 studies with 1,340 patients demonstrated that elevated PLR is associated with an increased mortality in patients diagnosed with several malignancies receiving various types of immune checkpoint inhibitors [39]. However, a relatively small meta-analysis from China, which included 4 studies with a total of 317 patients, found no association between overall survival and high PLR [40].

### NLPR and mortality

The association between NLPR levels and mortality has previously been described in the prediction of in-hospital mortality in septic patients and COVID-19 patients [41,42]. In these patients, a higher mortality risk was observed in patients with increased NLPR rates. To the best of our knowledge, the association between NLPR and mortality in patients undergoing immune checkpoint inhibitor therapy has not been reported previously. In our study, we found that the mortality risk was increased in the highest tertile and in the continuous analysis. The NLPR, which combines the NLR and PLR, may therefore be valuable as an addition to pretreatment diagnostics, providing a comprehensive index to reflect the inflammatory status of patients.

### SII and mortality

Elevated SII has been associated with increased mortality risk across various malignancies, including NSCLC, metastatic melanoma, renal cell cancer, biliary tract cancer, esophageal squamous cell carcinoma and pancreatic cancer [43–48]. It has been described that lower SII levels are associated with better survival outcomes, which aligns with the results in our study, were we observed that the highest tertiles of the SII was associated with an increased mortality risk.

### Explanation of association between hematological ratios and mortality

Based on previous studies, there could be several explanations for the association between hematological ratios and mortality. Elevated NLR levels reflect an increased neutrophil count or a decreased lymphocyte count, or both. Although the role of neutrophils in the tumor environment is not fully understood, it is believed that elevated neutrophil counts support angiogenesis and tumor growth by supporting pro-inflammatory chemokines and cytokines [49, 50]. In addition, a recent study found that the absence of neutrophils was associated with a reduction of metastases in breast cancer [51]. Not only neutrophils, but also platelets are thought to play a role in the tumor environment [52]. The interaction between platelets and tumor cells can result in adhering of tumor cells to the vascular endothelium, thereby playing a significant role in the development of metastasis [52]. The anti-tumor response, on the other hand, depends on lymphocyte infiltration in the tumor environment [52].

Increased SII values reflect higher neutrophil and platelet counts and lower lymphocyte count. NLR, PLR, NLPR and SII could therefore be a marker of imbalance between tumor promotion and tumor suppression, which can be in turn associated with higher mortality in patients with malignancy. In addition to tumor activity itself, indirect consequences of tumor activity may also influence hematological ratios and could be associated with mortality in this cohort, including treatment-related effects, bone marrow infiltration, infections and malnutrition in oncological patients [53–56].

### Strengths and limitations

The present study has several strengths. First, we used routine care data without exclusion criteria, thereby minimizing selection bias. Furthermore, utilizing routine care data reflects the different types of ICPi therapies in clinical practice. Nevertheless, this study has several limitations. First, there is uncertainty whether all registered irAE are in fact immune related. The registration and classification of irAE are performed by oncologists who could have falsely diagnosed irAE or could have missed irAE. However, determination and classification of irAE were conducted according the Common Terminology Criteria for Adverse Events [31]. Therefore, we consider it less likely that our results are biased by misclassification of irAE.

Secondly, we focused on analyzing linear associations, but it is possible that this method might overlook other types of associations. To minimize this risk, we also examined continuous risks in our analysis. Another limitation of our study was the lack of specification regarding the cause of mortality, as the cause of death among oncological patients in the Netherlands is generally not categorized with further specification of the exact cause. Therefore, we could not analyze the association between hematological ratios and cause-specific mortality, including infection or cardiovascular death. Finally, we had limited power to investigate the association between hematological ratios and outcomes for specific malignancy types.

### Clinical implications

The frequent development of irAE remains a significant challenge in the implementation of ICPi therapy. Our study reported that in 59.7 percent of patients receiving ICPi therapy irAE occurred. Our study findings did not show an association between hematological ratios and the development of irAE. Therefore, our study does not support the use of hematological ratios alone to predict irAE. However, our study showed increased mortality risks in patients with increased NLR, PLR, NLPR and SII ratios. Clinicians should be aware that patients with increased pretreatment NLR, PLR, NLPR and SII have higher mortality rates than patients with decreased pretreatment hematological ratios. Consequently, these pretreatment hematological ratios could be used as a tool to identify patients with an increased risk of mortality. However, future studies should investigate whether improvement of ratios or underlying diseases causing elevated ratios could influence the mortality risk of patients.

## Conclusion

In conclusion, we found an association between NLR, PLR, NLPR and SII and mortality. However, our findings did not show an association between elevated NLR, PLR, NLPR and SII and the development of irAE.
